# The 2020 Evidence-Based Promotion Ladder of Academic Plastic Surgery

**DOI:** 10.7759/cureus.15221

**Published:** 2021-05-24

**Authors:** Kevin M Klifto, Joseph Mellia, Alexander I Murphy, Fortunay Diatta, John P Fischer, Stephen J Kovach

**Affiliations:** 1 Plastic and Reconstructive Surgery, University of Missouri, Columbia, USA; 2 Plastic Surgery, University of Pennsylvania, Philadelphia, USA; 3 Plastic Surgery, Stony Brook University, Long Island, USA; 4 Plastic Surgery, Columbia University, New York, USA

**Keywords:** plastic surgery, academic rank, clinical faculty, plastic and reconstructive surgery, fellowship surgery, academic surgery, professor of surgery, plastic surgery residency, academic position, plastic surgery education

## Abstract

Background

Metrics were evaluated between academic plastic surgeons from different tiered training programs to determine promotion predictors within tiers and between tiers for those seeking promotion from assistant professor, associate professor, to full professors.

Methodology

We performed a retrospective, cross-sectional study by collecting 61 variables from full-time plastic surgery faculty affiliated with United States residency training programs during the 2020-2021 academic year. Surgeons were stratified into nine cohorts for comparison by professorship (assistant, associate, professor) and Doximity-ranked institution program tiers (Tier 1 = T1, Tier 2 = T2, Tier 3 = T3). Univariate followed by multivariate regressions with reciprocal transformation were performed to determine predictors more likely associated with promotion or lateral movement.

Results

A total of 98 programs listed 851 surgeons. T1/T2/T3 surgeon promotion predictors included more years in practice (p = 0.002; p < 0.001; p < 0.001) and greater number of last-author publications (p < 0.001; p < 0.001; p = 0.007). T1/T3 surgeon promotion predictors included higher h-indexes (p = 0.001; p = 0.002). T1 surgeon promotion predictors included being on journal editorial board (p = 0.040). T2 surgeon promotion predictors from assistant to associate included non-white race (p = 0.010). T3 surgeon promotion predictors included residency director (p = 0.009) and greater number of citations (p = 0.026). Promotion predictors from assistant, associate, and professors for T3/T2/T1 programs included greater number of last-author publications (p = 0.007; p = 0.002; p < 0.001). Movement from assistant and associate between T3/T2/T1 programs included plastic surgery department (p = 0.002; p < 0.001). Movement from assistant between programs included attending Top 10 US News medical schools (p = 0.012), attending more favorable Doximity-ranked research programs (p < 0.001), greater number of first-author publications (p = 0.017), and greater number of citations (p = 0.023). Movement from associate between programs included attending more favorable Doximity-ranked reputation programs (p = 0.017) and higher h-indexes (p = 0.017). Movement from professor between programs included receiving any American Association of Plastic Surgeons (AAPS) award (p = 0.039) and greater number of AAPS awards (p = 0.012).

Conclusions

Promotion predictors provided evidence to synthesize the Doximity-tiered *Promotion Ladder of Academic Plastic Surgery*.

## Introduction

Promotion in an academic surgical career is categorized by three subsequent stages of professorship. One typically starts his/her career as an assistant professor, advances through promotion to become an associate professor, and finally becomes a professor. Excellence is the basis for promotion demonstrated by five classic principles that define an academic surgical career. These principles include teaching, clinical productivity, administrative duty, community service, and research [1]. Teaching may be measured by trainee assessments and institutional recognition; clinical productivity may be measured by relative value units (RVUs) and income; administrative duties and community service may be measured by appointment to local, regional, and national board committees, societies, and/or associations; and research may be measured by the quality and number of peer-reviewed publications [1,2]. However, criteria for promotion often vary from one academic institution to another based on metrics implemented within each institution.

These differences often create challenges when comparing professorship and faculty metrics from one institution to another. Categorizing institutional programs with similar characteristics into a tiered system may clarify institution-specific metrics for academic plastic surgeons and provide promotional insight. Metrics can then be compared within each institutional tier to predict promotion from assistant professor to associate professor to full professor for a surgeon advancing his/her career within the same institution, and lateral movements among institutional tiers for a surgeon changing institutions. Identifying recent objective metrics from academic plastic surgeons across different institutional tiers may provide an updated guide for students, trainees, and junior faculty who plan to pursue and/or advance their careers through academic plastic surgery. The purpose of this study was to evaluate metrics among academic plastic surgeons from different training programs to determine predictors and guide prospective surgeons seeking promotion or changing institutions.

## Materials and methods

Study design

We performed an Institutional Review Board-exempt, retrospective, comparative, cross-sectional online review to collect data from full-time academic plastic surgeons affiliated with the United States Accreditation Council for Graduate Medical Education (ACGME)-accredited integrated and/or independent residency training programs during the 2020-2021 academic calendar year. Strengthening the Reporting of Observational Studies in Epidemiology (STROBE) guidelines were used throughout the review [3].

Study population

Plastic surgeons included full-time faculty from training programs available through the American Council of Academic Plastic Surgeons (ACAPS) website during the 2020-2021 academic calendar year. Each institution’s plastic surgery residency website was queried for faculty rosters. Non-plastic surgeons, adjunct faculty, and faculty without publicly available professorship statuses were excluded from the study.

Academic plastic surgeons were stratified into nine cohorts for comparison by professorship status (assistant, associate, professor) and institution program tier (Tier 1 = T1, Tier 2 = T2, Tier 3 = T3). Tiers were determined by publicly available Doximity plastic surgery program research rankings (rank 1-20 = T1, rank 21-50 = T2, rank >50/not ranked = T3) (Appendix 1). Doximity determined research metrics by collective h-indexes of program alumni. Research metrics were prioritized over reputation metrics to maximize objectivity [4].

Variables analyzed

A total of 61 publicly available variables were measured for each of the three professorship statuses and three program tiers based on full-time plastic surgeon faculty positions at ACGME-accredited institutions during the 2020-2021 academic year. Variables were manually searched and collected for each plastic surgeon from institutional websites, Doximity, LinkedIn, private-practice websites, organizational websites, research databases, and National Institutes of Health (NIH) websites from October 1, 2020 to November 19, 2020. A variable was considered if publicly available for each plastic surgeon. Four study members extracted study variables (KMK, JAM, AIM, FD). Following variable extraction, one of the four study members confirmed extracted variables for accuracy.

Variables included physician demographics (race, sex), current faculty academic institutions, medical degree backgrounds, advanced degrees, residency training, fellowship training, number of years in practice, program division or department status, residency and fellowship directorship, editorial board status, ACGME board membership, officer/director of the American Board of Plastic Surgeons (ABPS), regional society/association presidencies, national society/association presidencies, research metrics (Scopus; Reed Elsevier, London, United Kingdom), NIH grant funding, and American Association of Plastic Surgeons (AAPS) awards (Appendix 2).

Statistical analysis

Descriptive statistics were used to compare medians, interquartile ranges (IQR), ranges (minimum/maximum), odds ratios (OR), 95% confidence intervals (95% CI), area under the curves (AUC), frequencies, and percentages between variables based on the non-parametric population distribution assessed using the Shapiro-Wilk test. Analyses were performed to compare different professorship cohorts within the same tier (e.g., T1 assistant versus T1 associate versus T1 professor), followed by the same professorship cohorts between different institution tiers (e.g. T1 assistant versus T2 assistant versus T3 assistant). Dichotomous variables were assessed using Fisher’s exact cross-tabulation tests followed by the post-hoc Bonferroni tests with an α of 0.008 to determine which cohorts were different [5]. Continuous variables were assessed using Kruskal-Wallis tests followed by Dunn’s post-hoc tests. Univariate analyses were followed by multivariate stepwise logistic regressions using forward selection and reciprocal transformation to determine independent promotion predictors more likely associated with promotion or lateral movement. A receiver operating characteristic (ROC) curve was generated for each regression to assess the AUC for promotional predictor accuracy and discrimination. Analyses outcomes were two-tailed, with a significance level set at an α of 0.05. All analyses were performed using SPSS version 25.0 (IBM Corp., Armonk, NY).

## Results

A total of 951 full-time plastic surgeons were evaluated from 99 ACGME-accredited plastic surgery programs. Of the 951 plastic surgeons, 851 reported professorship status (assistants = 377, associates = 228, professors = 246) from 98 programs. Tier 1 programs included 114 assistants, 87 associates, and 97 professors. Tier 2 programs included 124 assistants, 61 associates, and 79 professors. Tier 3 programs included 139 assistants, 80 associates, and 70 professors. The following comparisons were performed to provide evidence for the Doximity-tiered *Promotion Ladder of Academic Plastic Surgery*: Tier 1 assistant, associate, and professor; Tier 2 assistant, associate, and professor; Tier 3 assistant, associate, and professor; assistant Tier 1, Tier 2, and Tier 3; associate Tier 1, Tier 2, and Tier 3; and professor Tier 1, Tier 2, and Tier 3.

Tier 1 assistant, associate, and professor

Univariate comparisons were performed to identify differences between T1 program assistant professors, associate professors, and professors (Table 1).

**Table 1:** Tier 1 comparisons between assistants, associates, and professors. MD: Doctor of Medicine; IMG: International Medical Graduate; DO: Doctor of Osteopathic medicine; US: United States; MA: Master of Arts; MS: Master of Science; MBA: Master of Business Administration; EMBA: Executive Master of Business Administration; MHS: Master of Health Science; MPH: Master of Public Health; PhD: Doctor of Philosophy; DDM: Doctor of Dental Medicine; DDS: Doctor of Dental Surgery; DMD: Doctor of Medicine in Dentistry; IQR: interquartile range; N/A: not applicable; ACGME: Accreditation Council for Graduate Medical Education; ABPS: American Board of Plastic Surgery; NIH: National Institutes of Health; AAPS: American Association of Plastic Surgeons Dichotomous variables were assessed using Fisher’s exact cross-tabulation tests followed by post-hoc Bonferroni tests with an α of 0.008 to determine which cohorts were different. Continuous variables were assessed using Kruskal-Wallis tests followed by Dunn’s post-hoc tests. *Statistically significant following post-hoc tests.

Variable		Assistant (n = 114)	Associate (n = 87)	Professor (n = 97)	P-value
Race, n (%)					0.074
	Non-white	42 (37)	34 (39)	24 (25)	
	White	72 (63)	53 (61)	73 (75)	
Sex, n (%)					<0.001
	Male	82 (72)	69 (79)	90 (93)*	
	Female	32 (28)	18 (21)	7 (7)*	
Medical degree, n (%)					
	MD	101 (89)	77 (89)	87 (90)	0.560
	IMG	13 (11)	9 (10)	9 (9)	0.891
	DO	0 (0)	1 (1)	1 (1)	0.615
Top 10 US News medical school, n (%)		22 (19)	26 (30)	26 (27)	0.170
US medical school, n (%)		101 (89)	78 (90)	88 (91)	0.891
Advanced degree, n (%)		25 (22)	20 (23)	20 (21)	0.920
Masters degree, n (%)		11 (10)	14 (16)	13 (13)	0.494
	MA	1 (1)	2 (2)	1 (1)	0.688
	MS/MSc	4 (4)	5 (6)	4 (4)	0.934
	MBA/EMBA	1 (1)	4 (5)	4 (4)	0.250
	MHS	1 (1)	0 (0)	3 (3)	0.198
	MPH	3 (3)	2 (2)	1 (1)	0.773
	Other	1 (1)	3 (3)	2 (2)	0.388
Doctorate degree, n (%)		14 (12)	7 (8)	10 (10)	0.643
	PhD	11 (10)	5 (6)	5 (5)	0.447
	DDM/DDS/DMD	3 (3)	2 (2)	4 (4)	0.762
	Other	0 (0)	0 (0)	0 (0)	---
Number of advanced degrees, median (range)		0 (0-3)	0 (0-2)	0 (0-2)	0.928
Residency program attended Doximity reputation rank, median (IQR)		14 (5-25)*	8 (4-20)	7 (4-16)*	0.014
Residency program attended Doximity research rank, median (IQR)		13 (6-27)	9 (6-17)	8 (4-15)	0.082
International residency attended, n (%)		4 (4)	3 (3)	6 (6)	0.633
US residency attended, n (%)					<0.001
	Integrated	57 (50)*	27 (31)	12 (12)*	
	Independent	55 (48)	56 (63)	81 (84)*	
	N/A	2 (2)	4 (5)	4 (4)	
Fellowships, n (%)		92 (81)	73 (84)	70 (72)	0.136
	Microsurgery	37 (32)	18 (21)	17 (18)	
	Hand	25 (22)	22 (25)	18 (19)	
	Craniofacial	23 (20)	22 (25)	28 (29)	
	Aesthetic	0 (0)	3 (3)	0 (0)	
	Burn	5 (4)	2 (1)	2 (2)	
	Peripheral nerve	0 (0)	1 (1)	1 (1)	
	Other	3 (3)	6 (7)	4 (4)	
	None	25 (22)	20 (23)	34 (35)	
International fellowship, n (%)		6 (5)	6 (7)	11 (11)	0.255
Research fellowship, n (%)		12 (11)	16 (18)	15 (15)	0.252
Number of fellowships, median (range)		1 (0-2)	1 (0-2)	1 (0-3)	0.235
Number of years in practice, median (range)		6 (0-38)*	13 (3-41)*	22 (5-46)*	<0.001
Department faculty, n (%)		38 (33)	35 (40)	26 (27)	0.157
Endowed status, n (%)		1 (1)	7 (8)	28 (29)	<0.001
Residency director, n (%)		6 (5)	5 (6)	7 (7)	0.826
Fellowship director, n (%)		5 (4)*	13 (15)	15 (15)	0.016
	Aesthetic	0 (0)	0 (0)	3 (3)	
	Craniofacial	3 (3)	4 (5)	5 (5)	
	Microsurgery	0 (0)	5 (6)	4 (4)	
	Hand	2 (2)	3 (3)	2 (2)	
	None	109 (96)	74 (85)	82 (85)	
Chief/chair, n (%)		1 (1)	1 (1)	20 (21)	<0.001
Former chief/chair, n (%)		0 (0)	0 (0)	5 (5)	0.017
Journal editorial board, n (%)		13 (11)	26 (30)	35 (36)	<0.001
ACGME board member, n (%)		0 (0)	0 (0)	5 (5)*	0.005
Officer/director of ABPS, n (%)		0 (0)*	1 (1)*	22 (23)*	<0.001
President of national society/association, n (%)		0 (0)*	1 (1)*	27 (28)*	<0.001
Number of presidencies of national society/association, median (range)		0 (0-0)	0 (0-1)	0 (0-6)*	<0.001
President of regional society/association, n (%)		0 (0)*	3 (3)	11 (11)	0.001
Total number of publications, median (range)		21 (1-160)*	55 (1-194)*	114 (1-920)*	<0.001
Number of first-author publications, median (range)		5 (0-72)*	10 (0-72)*	19 (0-159)*	<0.001
Number of last-author publications, median (range)		2 (0-44)*	14 (0-75)*	48 (0-542)*	<0.001
Number of first and last-author publications, median (range)		10 (0-100)*	24 (0-132)*	65 (0-701)*	<0.001
H-Index, median (range)		8 (0-27)*	14 (0-41)*	26 (1-104)*	<0.001
Number of citations, median (range)		243 (0-4,226)*	795 (0-15,520)*	2611 (1-42,005)*	<0.001
NIH funded, n (%)		6 (5)*	12 (14)*	33 (34)*	<0.001
Number of NIH grants, median (range)		0 (0-6)	0 (0-12)	0 (0-89)*	<0.001
Total NIH funding, median (range)		0 (0-1,770,546)	0 (0-9,645,674)	0 (0-42,232,565)*	<0.001
AAPS award, n (%)		8 (7)	4 (5)	27 (28)*	<0.001
Number of distinct AAPS awards, median (range)		0 (0-1)	0 (0-1)	0 (0-4)*	<0.001

Following multivariate analysis, plastic surgeons were more likely to be promoted from assistants to associates and professors with more years in practice (OR: 1.04, 95% CI: 1.02, 1.07; AUC: 0.72; p = 0.002), if on a journal editorial board (OR: 1.87, 95% CI: 1.03, 3.40; AUC: 0.73; p = 0.040), a greater number of last-author publications (OR: 1.07, 95% CI: 1.05, 1.08; AUC: 0.64; p < 0.001), and higher h-indexes (OR: 1.08, 95% CI: 1.03, 1.13; AUC: 0.73; p = 0.001). Plastic surgeons were as likely to be promoted to associates and professors with a greater number of citations (OR: 1.00, 95% CI: 1.00, 1.00; AUC: 0.52; p = 0.014).

Tier 2 assistant, associate, and professor

Univariate comparisons were performed to identify differences between T2 program assistant professors, associate professors, and professors (Table 2).

**Table 2:** Tier 2 comparisons between assistants, associates, and professors. MD: Doctor of Medicine; IMG: International Medical Graduate; DO: Doctor of Osteopathic medicine; US: United States; MA: Master of Arts; MS: Master of Science; MBA: Master of Business Administration; EMBA: Executive Master of Business Administration; MHS: Master of Health Science; MPH: Master of Public Health; PhD: Doctor of Philosophy; DDM: Doctor of Dental Medicine; DDS: Doctor of Dental Surgery; DMD: Doctor of Medicine in Dentistry; IQR: interquartile range; N/A: not applicable; ACGME: Accreditation Council for Graduate Medical Education; ABPS: American Board of Plastic Surgery; NIH: National Institutes of Health; AAPS: American Association of Plastic Surgeons Dichotomous variables were assessed using Fisher’s exact cross-tabulation tests followed by post-hoc Bonferroni tests with an α of 0.008 to determine which cohorts were different. Continuous variables were assessed using Kruskal-Wallis tests followed by Dunn’s post-hoc tests. *Statistically significant following post-hoc tests.

Variable		Assistant (n=124)	Associate (n=61)	Professor (n=79)	p-value
Race, n (%)					0.014
	Non-white	36 (29)	30 (49)*	22 (28)	
	White	88 (71)	31 (51)	57 (72)	
Sex, n (%)					<0.001
	Male	83 (67)	45 (74)	71 (90)*	
	Female	41 (33)	16 (26)	8 (10)*	
Medical degree, n (%)					
	MD	113 (91)	51 (84)	58 (73)	0.195
	IMG	9 (7)*	10 (16)*	21 (27)*	0.001
	DO	2 (2)	0 (0)	0 (0)	0.721
Top 10 US News medical school, n (%)		18 (15)	5 (8)	11 (14)	0.480
US medical school, n (%)		115 (93)*	51 (84)	58 (73)	0.001
Advanced degree, n (%)		18 (15)	13 (21)	16 (20)	0.384
Masters degree, n (%)		11 (9)	4 (7)	7 (9)	0.880
	MA	0 (0)	1 (2)	0 (0)	0.233
	MS/MSc	5 (4)	2 (3)	2 (3)	0.910
	MBA/EMBA	3 (2)	2 (3)	5 (6)	0.313
	MHS	0 (0)	0 (0)	0 (0)	---
	MPH	2 (2)	0 (0)	0 (0)	0.721
	Other	1 (1)	0 (0)	0 (0)	0.999
Doctorate degree, n (%)		7 (6)	9 (15)	9 (11)	0.080
	PhD	7 (6)	5 (8)	5 (6)	0.724
	DDM/DDS/DMD	0 (0)	3 (5)	4 (5)	0.016
	Other	0 (0)	0 (0)	0 (0)	---
Number of advanced degrees, median (range)		0 (0-1)	0 (0-1)	0 (0-1)	0.392
Residency program attended Doximity reputation rank, median (IQR)		23 (10-45)*	31 (17-49)	16 (6-33)*	0.005
Residency program attended Doximity research rank, median (IQR)		32 (12-46)	29 (20-43)	22 (8-41)	0.182
International residency attended, n (%)		3 (2)	6 (10)	13 (16)	0.001
US residency attended, n (%)					<0.001
	Integrated	69 (56)*	17 (28)	11 (14)*	
	Independent	51 (41)*	39 (64)	59 (75)*	
	N/A	4 (3)	5 (8)	9 (11)	
Fellowships, n (%)		103 (83)	54 (89)	57 (74)	0.087
	Microsurgery	26 (21)	13 (21)	10 (13)	
	Hand	49 (40)	18 (30)	27 (34)	
	Craniofacial	23 (19)	15 (25)	14 (18)	
	Aesthetic	2 (2)	6 (10)	2 (3)	
	Burn	2 (2)	3 (5)	3 (4)	
	Peripheral nerve	1 (1)	0 (0)	3 (4)	
	Other	3 (2)	1 (2)	5 (6)	
	None	24 (19)	10 (16)	25 (32)	
International fellowship, n (%)		10 (8)	4 (7)	7 (9)	0.916
Research fellowship, n (%)		16 (13)	9 (15)	21 (27)*	0.039
Number of fellowships, median (range)		1 (0-4)	1 (0-5)	1 (0-3)	0.906
Number of years in practice, median (range)		4 (0-24)*	12 (5-44)*	26 (8-51)*	<0.001
Department faculty, n (%)		29 (23)	17 (28)	25 (32)	0.415
Endowed status, n (%)		0 (0)	0 (0)	18 (23)*	<0.001
Residency director, n (%)		8 (6)	10 (16)	9 (12)	0.096
Fellowship director, n (%)		6 (5)	4 (7)	10 (13)*	<0.001
	Aesthetic	0 (0)	0 (0)	1 (1)	
	Craniofacial	1 (1)	1 (2)	4 (5)	
	Microsurgery	3 (2)	2 (3)	1 (1)	
	Hand	2 (2)	1 (2)	4 (5)	
	None	118 (95)	57 (93)	67 (87)	
Chief/chair, n (%)		1 (1)	5 (8)	22 (28)*	<0.001
Former chief/chair, n (%)		0 (0)	0 (0)	8 (10)*	<0.001
Journal editorial board, n (%)		19 (15)	10 (16)	30 (38)*	0.001
ACGME board member, n (%)		0 (0)	0 (0)	3 (4)*	0.038
Officer/director of ABPS, n (%)		0 (0)*	2 (3)	19 (25)*	<0.001
President of national society/association, n (%)		0 (0)*	3 (5)	22 (29)*	<0.001
Number of presidencies of national society/association, median (range)		0 (0-0)	0 (0-1)	0 (0-5)*	0.013
President of regional society/association, n (%)		0 (0)*	0 (0)	9 (12)*	<0.001
Number of publications, median (range)		10 (0-110)*	30 (0-139)*	64 (2-625)*	<0.001
Number of first author publications, median (range)		4 (0-28)	4 (0-68)	14 (0-115)*	<0.001
Number of last author publications, median (range)		1 (0-44)*	9 (0-45)*	23 (1-188)*	<0.001
Number of first and last author publications, median (range)		5 (0-63)*	15 (0-101)*	36 (2-303)*	<0.001
H-Index, median (range)		5 (0-20)*	11 (0-23)*	20 (0-81)*	<0.001
Number of citations, median (range)		85 (0-1839)*	459 (0-2787)*	1487 (0-24114)*	<0.001
NIH funded, n (%)		3 (2)*	7 (11)	12 (15)	0.001
Number of NIH grants, median (range)		0 (0-5)*	0 (0-9)	0 (0-33)*	0.003
Total NIH Funding, median (range)		0 (0-717332)*	0 (0-12829230)	0 (0-13597384)*	0.006
AAPS award, n (%)		3 (2)	1 (2)	9 (11)*	0.010
Number of distinct AAPS awards, median (range)		0 (0-1)	0 (0-1)	0 (0-4)	0.732

Following multivariate analysis, plastic surgeons were more likely to be promoted from assistants to associates if non-white (OR: 2.76, 95% CI: 1.27, 5.99; AUC: 0.64; p = 0.010). Plastic surgeons were more likely to be promoted from assistants to associates and professors with more years in practice (OR: 1.22, 95% CI: 1.15, 1.29; AUC: 0.67; p < 0.001) and a greater number of last-author publications (OR: 1.13, 95% CI: 1.09, 1.17; AUC: 0.72; p < 0.001).

Tier 3 Assistant, Associate, and Professor

Univariate comparisons were performed to identify differences between T3 program assistant professors, associate professors, and professors (Table 3).

**Table 3:** Tier 3 comparisons between assistants, associates, and professors. MD: Doctor of Medicine; IMG: International Medical Graduate; DO: Doctor of Osteopathic medicine; US: United States; MA: Master of Arts; MS: Master of Science; MBA: Master of Business Administration; EMBA: Executive Master of Business Administration; MHS: Master of Health Science; MPH: Master of Public Health; PhD: Doctor of Philosophy; DDM: Doctor of Dental Medicine; DDS: Doctor of Dental Surgery; DMD: Doctor of Medicine in Dentistry; IQR: interquartile range; N/A: not applicable; ACGME: Accreditation Council for Graduate Medical Education; ABPS: American Board of Plastic Surgery; NIH: National Institutes of Health; AAPS: American Association of Plastic Surgeons Dichotomous variables were assessed using Fisher’s exact cross-tabulation tests followed by post-hoc Bonferroni tests with an α of 0.008 to determine which cohorts were different. Continuous variables were assessed using Kruskal-Wallis tests followed by Dunn’s post-hoc tests. *Statistically significant following post-hoc tests.

Variable		Assistant (n = 139)	Associate (n = 80)	Professor (n = 70)	P-value
Race, n (%)					0.445
	Non-white	50 (36)	23 (29)	20 (29)	
	White	89 (64)	57 (71)	50 (71)	
Sex, n (%)					0.003
	Male	97 (70)*	69 (86)	61 (87)	
	Female	42 (30)*	11 (14)	9 (13)	
Medical degree, n (%)					
	MD	115 (83)	67 (84)	58 (83)	0.733
	IMG	22 (16)	13 (16)	12 (17)	0.978
	DO	2 (1)	0 (0)	0 (0)	0.499
Top 10 US News medical school, n (%)		7 (5)	11 (14)	13 (19)	0.005
US medical school, n (%)		117 (84)	67 (84)	58 (83)	0.978
Advanced degree, n (%)		19 (14)	18 (23)	14 (20)	0.198
Masters degree, n (%)		12 (9)	8 (10)	9 (13)	0.723
	MA	3 (2)	0 (0)	1 (1)	0.580
	MS/MSc	0 (0)	2 (3)	5 (7)	0.079
	MBA/EMBA	4 (3)	4 (5)	3 (4)	0.671
	MHS	0 (0)	0 (0)	0 (0)	0.499
	MPH	0 (0)	1 (1)	1 (1)	0.999
	Other	0 (0)	1 (1)	1 (1)	0.366
Doctorate degree, n (%)		7 (5)	11 (14)*	6 (9)	0.038
	PhD	6 (4)	7 (9)	4 (6)	0.375
	DDM/DDS/DMD	0 (0)	5 (6)*	0 (0)	0.006
	Other	1 (1)	0 (0)	0 (0)	0.499
Number of advanced degrees, median (range)		0 (0-1)	0 (0-2)	0 (0-2)	0.182
Residency program attended Doximity reputation rank, median (IQR)		47 (12-64)	32 (16-61)	28 (9-53)	0.090
Residency program attended Doximity research rank, median (IQR)		45 (16-65)	31 (9-63)	27 (12-55)	0.113
International residency attended, n (%)		5 (4)	4 (5)	4 (6)	0.760
US residency attended, n (%)					0.002
	Integrated	46 (33)*	17 (21)	8 (12)*	
	Independent	88 (63)*	58 (73)	59 (84)*	
	N/A	5 (4)	5 (6)	3 (4)	
Fellowships, n (%)		99 (71)	62 (78)	46 (66)	0.271
	Microsurgery	37 (27)	14 (18)	8 (12)	
	Hand	28 (20)	24 (30)	11 (16)	
	Craniofacial	28 (20)	21 (26)	17 (24)	
	Aesthetic	3 (2)	3 (4)	3 (4)	
	Burn	6 (4)	1 (1)	3 (4)	
	Peripheral nerve	1 (1)	0 (0)	1 (1)	
	Other	4 (3)	2 (3)	3 (4)	
	None	45 (32)	21 (26)	28 (40)	
International fellowship, n (%)		8 (6)	7 (9)	6 (9)	0.614
Research fellowship, n (%)		14 (10)	8 (10)	12 (17)	0.291
Number of fellowships, median (range)		1 (0-4)	1 (0-4)	1 (0-3)	0.607
Number of years in practice, median (range)		5 (0-37)*	14 (0-46)*	27 (7-50)*	<0.001
Department faculty, n (%)		24 (17)	6 (8)	10 (14)	0.118
Endowed status, n (%)		0 (0)	0 (0)	9 (13)*	<0.001
Residency director, n (%)		9 (6)	16 (20)*	13 (19)*	0.004
Fellowship director, n (%)		4 (3)	3 (4)	5 (7)	0.323
	Aesthetic	0 (0)	1 (1)	1 (1)	
	Craniofacial	2 (1)	1 (1)	2 (3)	
	Microsurgery	2 (1)	0 (0)	2 (3)	
	Hand	0 (0)	1 (1)	0 (0)	
	None	135 (97)	77 (96)	65 (93)	
Chief/chair, n (%)		1 (1)*	12 (15)*	30 (43)*	<0.001
Former chief/chair, n (%)		0 (0)	0 (0)	2 (3)	0.058
Journal editorial board, n (%)		14 (10)	10 (13)	18 (26)*	0.012
ACGME board member, n (%)		0 (0)	0 (0)	1 (1)	0.242
Officer/director of ABPS, n (%)		0 (0)*	1 (1)	11 (16)*	<0.001
President of national society/association, n (%)		0 (0)*	3 (4)	9 (13)*	<0.001
Number of presidencies of national society/association, median (range)		0 (0-0)	0 (0-1)	0 (0-4)*	<0.001
President of regional society/association, n (%)		0 (0)*	2 (3)	7 (10)*	<0.001
Number of publications, median (range)		8 (0-117)*	19 (1-214)*	36 (0-185)*	<0.001
Number of first-author publications, median (range)		2 (0-32)*	4 (0-43)*	8 (0-41)*	<0.001
Number of last-author publications, median (range)		1 (0-33)*	5 (0-112)*	12 (0-124)*	<0.001
Number of first and last-author publications, median (range)		3 (0-65)*	8 (0-138)*	23 (0-136)*	<0.001
H-Index, median (range)		4 (0-42)*	8 (0-27)*	15 (0-43)*	<0.001
Number of citations, median (range)		94 (0-6546)*	246 (0-2958)*	884 (0-5447)*	<0.001
NIH funded, n (%)		2 (1)	4 (5)	4 (6)	0.146
Number of NIH grants, median (range)		0 (0-5)	0 (0-9)	0 (0-10)	0.191
Total NIH funding, median (range)		0 (0-434,867)	0 (0-2,268,076)	0 (0-2,971,116)	0.183
AAPS award, n (%)		0 (0)*	2 (3)*	6 (9)*	0.003
Number of distinct AAPS awards, median (range)		0 (0-0)	0 (0-1)	0 (0-5)*	0.010

Following multivariate analysis, plastic surgeons were more likely to be promoted from assistants to associates and professors with more years in practice (OR: 1.12, 95% CI: 1.09, 1.16; AUC: 0.69; p < 0.001), being a residency director (OR: 2.81, 95% CI: 1.30, 6.08; AUC: 0.74; p = 0.009), a greater number of last-author publications (OR: 1.09, 95% CI: 1.05, 1.13; AUC: 0.75; p < 0.001), higher h-indexes (OR: 1.17, 95% CI: 1.06, 1.30; AUC: 0.74; p = 0.002), and a greater number of citations (OR: 1.01, 95% CI: 1.00, 1.02; AUC: 0.26; p = 0.026).

Assistant Tier 1, Tier 2, and Tier 3

Univariate comparisons were performed to identify differences between T1 assistant professors, T2 assistant professors, and T3 assistant professors (Table 4).

**Table 4:** Assistant professor comparisons between Tier 1, Tier 2, and Tier 3 programs. MD: Doctor of Medicine; IMG: International Medical Graduate; DO: Doctor of Osteopathic medicine; US: United States; MA: Master of Arts; MS: Master of Science; MBA: Master of Business Administration; EMBA: Executive Master of Business Administration; MHS: Master of Health Science; MPH: Master of Public Health; PhD: Doctor of Philosophy; DDM: Doctor of Dental Medicine; DDS: Doctor of Dental Surgery; DMD: Doctor of Medicine in Dentistry; IQR: interquartile range; N/A: not applicable; ACGME: Accreditation Council for Graduate Medical Education; ABPS: American Board of Plastic Surgery; NIH: National Institutes of Health; AAPS: American Association of Plastic Surgeons Dichotomous variables were assessed using Fisher’s exact cross-tabulation tests followed by post-hoc Bonferroni tests with an α of 0.008 to determine which cohorts were different. Continuous variables were assessed using Kruskal-Wallis tests followed by Dunn’s post-hoc tests. *Statistically significant following post-hoc tests.

Variable		Tier 1 Assistant (n = 114)	Tier 2 Assistant (n = 124)	Tier 3 Assistant (n = 139)	P-value
Race, n (%)					0.113
	Non-white	42 (37)	36 (29)	50 (36)	
	White	72 (63)	88 (71)	89 (64)	
Sex, n (%)					0.704
	Male	82 (72)	83 (67)	97 (70)	
	Female	32 (28)	41 (33)	42 (30)	
Medical degree, n (%)					
	MD	101 (89)	113 (91)	77 (89)	0.349
	IMG	13 (11)	9 (7)	9 (10)	0.103
	DO	0 (0)	2 (2)	1 (1)	0.556
Top 10 US News medical school, n (%)		22 (19)*	18 (15)*	7 (5)	0.001
US medical school, n (%)		101 (89)	115 (93)	117 (84)	0.103
Advanced degree, n (%)		25 (22)	18 (15)	19 (14)	0.178
Masters degree, n (%)		11 (10)	11 (9)	12 (9)	0.999
	MA	1 (1)	0 (0)	3 (2)	0.328
	MS/MSc	4 (4)	5 (4)	0 (0)	0.402
	MBA/EMBA	1 (1)	3 (2)	4 (3)	0.606
	MHS	1 (1)	0 (0)	0 (0)	0.644
	MPH	3 (3)	2 (2)	0 (0)	0.451
	Other	1 (1)	1 (1)	0 (0)	0.999
Doctorate degree, n (%)		14 (12)	7 (6)	7 (5)	0.047
	PhD	11 (10)	7 (6)	6 (4)	0.247
	DDM/DDS/DMD	3 (3)*	0 (0)	0 (0)	0.027
	Other	0 (0)	0 (0)	1 (1)	0.644
Number of advanced degrees, median (range)		0 (0-3)	0 (0-1)	0 (0-1)	0.156
Residency program attended Doximity reputation rank, median (IQR)		14 (5-25)*	23 (10-45)*	47 (12-64)*	<0.001
Residency program attended Doximity research rank, median (IQR)		13 (6-27)*	32 (12-46)*	45 (16-65)*	<0.001
International residency attended, n (%)		4 (4)	3 (2)	5 (4)	0.827
US residency attended, n (%)					0.002
	Integrated	57 (50)	69 (56)*	46 (33)*	
	Independent	55 (48)	51 (41)*	88 (63)*	
	N/A	2 (2)	4 (3)	5 (4)	
Fellowships, n (%)		92 (81)	103 (83)	99 (71)	0.052
	Microsurgery	37 (32)	26 (21)	37 (27)	
	Hand	25 (22)	49 (40)	28 (20)	
	Craniofacial	23 (20)	23 (19)	28 (20)	
	Aesthetic	0 (0)	2 (2)	3 (2)	
	Burn	5 (4)	2 (2)	6 (4)	
	Peripheral nerve	0 (0)	1 (1)	1 (1)	
	Other	3 (3)	3 (2)	4 (3)	
	None	25 (22)	24 (19)	45 (32)	
International fellowship, n (%)		6 (5)	10 (8)	8 (6)	0.637
Research fellowship, n (%)		12 (11)	16 (13)	14 (10)	0.938
Number of fellowships, median (range)		1 (0-2)	1 (0-4)	1 (0-4)	0.109
Number of years in practice, median (range)		6 (0-38)	4 (0-24)	5 (0-37)	0.095
Department faculty, n (%)		38 (33)*	29 (23)	24 (17)*	0.012
Endowed status, n (%)		1 (1)	0 (0)	0 (0)	0.302
Residency director, n (%)		6 (5)	8 (6)	9 (6)	0.928
Fellowship director, n (%)		5 (4)	6 (5)	4 (3)	0.718
	Aesthetic	0 (0)	0 (0)	0 (0)	
	Craniofacial	3 (3)	1 (1)	2 (1)	
	Microsurgery	0 (0)	3 (2)	2 (1)	
	Hand	2 (2)	2 (2)	0 (0)	
	None	109 (96)	118 (95)	135 (97)	
Chief/chair, n (%)		1 (1)	1 (1)	1 (1)	0.999
Former chief/chair, n (%)		0 (0)	0 (0)	0 (0)	---
Journal editorial board, n (%)		13 (11)	19 (15)	14 (10)	0.451
ACGME board member, n (%)		0 (0)	0 (0)	0 (0)	---
Officer/director of ABPS, n (%)		0 (0)	0 (0)	0 (0)	---
President of national society/association, n (%)		0 (0)	0 (0)	0 (0)	---
Number of presidencies of national society/association, median (range)		0 (0-0)	0 (0-0)	0 (0-0)	0.999
President of regional society/association, n (%)		0 (0)	0 (0)	0 (0)	---
Number of publications, median (range)		21 (1-160)	10 (0-110)	8 (0-117)*	<0.001
Number of first-author publications, median (range)		5 (0-72)	4 (0-28)	2 (0-32)*	<0.001
Number of last-author publications, median (range)		2 (0-44)	1 (0-44)	1 (0-33)*	<0.001
Number of first and last-author publications, median (range)		10 (0-100)*	5 (0-63)*	3 (0-65)*	<0.001
H-Index, median (range)		8 (0-27)	5 (0-20)	4 (0-42)*	<0.001
Number of citations, median (range)		243 (0-4,226)	85 (0-1,839)	94 (0-6,546)*	<0.001
NIH funded, n (%)		6 (5)	3 (2)	2 (1)	0.216
Number of NIH grants, median (range)		0 (0-6)	0 (0-5)	0 (0-5)	0.186
Total NIH funding, median (range)		0 (0-1,770,546)	0 (0-717,332)	0 (0-434,867)	0.179
AAPS award, n (%)		8 (7)*	3 (2)	0 (0)*	0.002
Number of distinct AAPS awards, median (range)		0 (0-1)	0 (0-1)	0 (0-0)*	0.004

Following multivariate analysis, plastic surgeons were more likely to move laterally from an assistant at a T3 program to a T2 program and T1 program if they attended a Top 10 US News medical school (OR: 2.30, 95% CI: 1.21, 4.41; AUC: 0.56; p = 0.012), if they attended a more favorable Doximity-ranked research program (OR: 1.03, 95% CI: 1.01, 1.04; AUC: 0.28; p < 0.001), with a greater number of first-author publications (OR: 1.04, 95% CI: 1.01, 1.08; AUC: 0.73; p = 0.017), with a greater number of last-author publications (OR: 1.05, 95% CI: 1.01, 1.09; AUC: 0.86; p = 0.007), if they had a greater number of citations (OR: 1.01, 95% CI: 1.00, 1.02; AUC: 0.78; p = 0.023), and if plastic surgery was a department at T2 and T1 programs (OR: 2.29, 95% CI: 1.37, 3.83; AUC: 0.56; p = 0.002).

Associate Tier 1, Tier 2, and Tier 3

Univariate comparisons were performed to identify differences between T1 program associate professors, T2 program associate professors, and T3 program associate professors (Table 5).

**Table 5:** Associate professor comparisons between Tier 1, Tier 2, and Tier 3 programs. MD: Doctor of Medicine; IMG: International Medical Graduate; DO: Doctor of Osteopathic medicine; US: United States; MA: Master of Arts; MS: Master of Science; MBA: Master of Business Administration; EMBA: Executive Master of Business Administration; MHS: Master of Health Science; MPH: Master of Public Health; PhD: Doctor of Philosophy; DDM: Doctor of Dental Medicine; DDS: Doctor of Dental Surgery; DMD: Doctor of Medicine in Dentistry; IQR: interquartile range; N/A: not applicable; ACGME: Accreditation Council for Graduate Medical Education; ABPS: American Board of Plastic Surgery; NIH: National Institutes of Health; AAPS: American Association of Plastic Surgeons Dichotomous variables were assessed using Fisher’s exact cross-tabulation tests followed by post-hoc Bonferroni tests with an α of 0.008 to determine which cohorts were different. Continuous variables were assessed using Kruskal-Wallis tests followed by Dunn’s post-hoc tests. *Statistically significant following post-hoc tests.

Variable		Tier 1 Associate (n = 87)	Tier 2 Associate (n = 61)	Tier 3 Associate (n = 80)	P-value
Race, n (%)					0.047
	Non-white	34 (39)	30 (49)*	23 (29)	
	White	53 (61)	31 (51)	57 (71)	
Sex, n (%)					0.187
	Male	69 (79)	45 (74)	69 (86)	
	Female	18 (21)	16 (26)	11 (14)	
Medical degree, n (%)					
	MD	77 (89)	51 (84)	67 (84)	---
	IMG	9 (10)	10 (16)	13 (16)	0.470
	DO	1 (1)	0 (0)	0 (0)	---
Top 10 US News medical school, n (%)		26 (30)*	5 (8)	11 (14)	0.002
US medical school, n (%)		78 (90)	51 (84)	67 (84)	0.357
Advanced degree, n (%)		20 (23)	13 (21)	18 (23)	0.999
Masters degree, n (%)		14 (16)	4 (7)	8 (10)	0.260
	MA	2 (2)	1 (2)	1 (1)	0.489
	MS/MSc	5 (6)	2 (3)	5 (7)	0.900
	MBA/EMBA	4 (5)	2 (3)	3 (4)	0.923
	MHS	0 (0)	0 (0)	0 (0)	---
	MPH	2 (2)	0 (0)	1 (1)	0.782
	Other	3 (3)	0 (0)	1 (1)	0.456
Doctorate degree, n (%)		7 (8)	9 (15)	11 (14)	0.389
	PhD	5 (6)	5 (8)	7 (9)	0.773
	DDM/DDS/DMD	2 (2)	3 (5)	5 (6)	0.472
	Other	0 (0)	0 (0)	0 (0)	---
Number of advanced degrees, median (range)		0 (0-2)	0 (0-1)	0 (0-2)	0.961
Residency program attended Doximity reputation rank, median (IQR)		8 (4-20)*	31 (17-49)	32 (16-61)	<0.001
Residency program attended Doximity research rank, median (IQR)		9 (6-17)*	29 (20-43)	31 (9-63)	<0.001
International residency attended, n (%)		3 (3)	6 (10)	4 (5)	0.318
US residency attended, n (%)					0.458
	Integrated	27 (31)	17 (28)	17 (21)	
	Independent	56 (63)	39 (64)	58 (73)	
	N/A	4 (5)	5 (8)	5 (6)	
Fellowships, n (%)		73 (84)	54 (89)	62 (78)	0.240
	Microsurgery	18 (21)	13 (21)	14 (18)	
	Hand	22 (25)	18 (30)	24 (30)	
	Craniofacial	22 (25)	15 (25)	21 (26)	
	Aesthetic	3 (3)	6 (10)	3 (4)	
	Burn	2 (1)	3 (5)	1 (1)	
	Peripheral nerve	1 (1)	0 (0)	0 (0)	
	Other	6 (7)	1 (2)	2 (3)	
	None	20 (23)	10 (16)	21 (26)	
International fellowship, n (%)		6 (7)	4 (7)	7 (9)	0.902
Research fellowship, n (%)		16 (18)	9 (15)	8 (10)	0.297
Number of fellowships, median (range)		1 (0-2)	1 (0-5)	1 (0-4)	0.233
Number of years in practice, median (range)		13 (3-41)	12 (5-44)	14 (0-46)	0.893
Department faculty, n (%)		35 (40)*	17 (28)*	6 (8)*	<0.001
Endowed status, n (%)		7 (8)*	0 (0)	0 (0)	0.003
Residency director, n (%)		5 (6)*	10 (16)	16 (20)	0.016
Fellowship director, n (%)		13 (15)	4 (7)	3 (4)	0.064
	Aesthetic	0 (0)	0 (0)	1 (1)	
	Craniofacial	4 (5)	1 (2)	1 (1)	
	Microsurgery	5 (6)	2 (3)	0 (0)	
	Hand	3 (3)	1 (2)	1 (1)	
	None	74 (85)	57 (93)	77 (96)	
Chief/chair, n (%)		1 (1)*	5 (8)*	12 (15)*	0.002
Former chief/chair, n (%)		0 (0)	0 (0)	0 (0)	---
Journal editorial board, n (%)		26 (30)*	10 (16)	10 (13)	0.024
ACGME board member, n (%)		0 (0)	0 (0)	0 (0)	---
Officer/director of ABPS, n (%)		1 (1)	2 (3)	1 (1)	0.681
President of national society/association, n (%)		1 (1)	3 (5)	3 (4)	0.386
Number of presidencies of national society/association, median (range)		0 (0-1)	0 (0-1)	0 (0-1)	0.388
President of regional society/association, n (%)		3 (3)	0 (0)	2 (3)	0.445
Number of publications, median (range)		55 (1-194)	30 (0-139)	19 (1-214)*	<0.001
Number of first-author publications, median (range)		10 (0-72)*	4 (0-68)	4 (0-43)	<0.001
Number of last-author publications, median (range)		14 (0-75)*	9 (0-45)	5 (0-112)	<0.001
Number of first and last-author publications, median (range)		24 (0-132)*	15 (0-101)	8 (0-138)	<0.001
H-Index, median (range)		14 (0-41)	11 (0-23)	8 (0-47)*	<0.001
Number of citations, median (range)		795 (0-15,520)	459 (0-2,787)	249 (0-8,692)*	<0.001
NIH funded, n (%)		12 (14)	7 (11)	4 (5)	0.130
Number of NIH grants, median (range)		0 (0-12)	0 (0-9)	0 (0-9)	0.136
Total NIH funding, median (range)		0 (0-9,645,674)	0 (0-12,829,230)	0 (0-2,268,076)	0.133
AAPS award, n (%)		4 (5)	1 (2)	3 (4)	0.745
Number of distinct AAPS awards, median (range)		0 (0-1)	0 (0-1)	0 (0-5)	0.625

Following multivariate analysis, plastic surgeons were more likely to move laterally from an associate at a T3 program to a T2 program and T1 program if they attended a more favorable Doximity-ranked reputation program (OR: 1.02, 95% CI: 1.00, 1.04; AUC: 0.23; p = 0.017), a greater number of last-author publications (OR: 1.03, 95% CI: 1.01, 1.05; AUC: 0.72; p = 0.002), if they had higher h-indexes (OR: 1.12, 95% CI: 1.02, 1.24; AUC: 0.76; p = 0.017), and if plastic surgery was a department at T2 and T1 programs (OR: 4.24, 95% CI: 2.01, 8.93; AUC: 0.63; p < 0.001).

Professor Tier 1, Tier 2, and Tier 3

Univariate comparisons were performed to identify differences between T1 program professors, T2 program professors, and T3 program professors (Table 6).

**Table 6:** Professor comparisons between Tier 1, Tier 2, and Tier 3 programs. MD: Doctor of Medicine; IMG: International Medical Graduate; DO: Doctor of Osteopathic medicine; US: United States; MA: Master of Arts; MS: Master of Science; MBA: Master of Business Administration; EMBA: Executive Master of Business Administration; MHS: Master of Health Science; MPH: Master of Public Health; PhD: Doctor of Philosophy; DDM: Doctor of Dental Medicine; DDS: Doctor of Dental Surgery; DMD: Doctor of Medicine in Dentistry; IQR: interquartile range; N/A: not applicable; ACGME: Accreditation Council for Graduate Medical Education; ABPS: American Board of Plastic Surgery; NIH: National Institutes of Health; AAPS: American Association of Plastic Surgeons Dichotomous variables were assessed using Fisher’s exact cross-tabulation tests followed by post-hoc Bonferroni tests with an α of 0.008 to determine which cohorts were different. Continuous variables were assessed using Kruskal-Wallis tests followed by Dunn’s post-hoc tests. *Statistically significant following post-hoc tests.

Variable		Tier 1 Professor (n = 97)	Tier 2 Professor (n = 79)	Tier 3 Professor (n = 70)	P-value
Race, n (%)					0.856
	Non-white	24 (25)	22 (28)	20 (29)	
	White	73 (75)	57 (72)	50 (71)	
Sex, n (%)					0.511
	Male	90 (93)	71 (90)	61 (87)	
	Female	7 (7)	8 (10)	9 (13)	
Medical degree, n (%)					
	MD	87 (90)	58 (73)	58 (83)	0.632
	IMG	9 (9)	21 (27)*	12 (17)	0.009
	DO	1 (1)	0 (0)	0 (0)	0.999
Top 10 US News medical school, n (%)		26 (27)	11 (14)	13 (19)	0.105
US medical school, n (%)		88 (91)	58 (73)*	58 (83)	0.009
Advanced degree, n (%)		20 (21)	16 (20)	14 (20)	0.999
Masters degree, n (%)		13 (13)	7 (9)	9 (13)	0.766
	MA	1 (1)	0 (0)	1 (1)	0.749
	MS/MSc	4 (4)	2 (3)	5 (7)	0.422
	MBA/EMBA	4 (4)	5 (6)	3 (4)	0.546
	MHS	3 (3)	0 (0)	0 (0)	0.115
	MPH	1 (1)	0 (0)	1 (1)	0.749
	Other	2 (2)	0 (0)	1 (1)	0.468
Doctorate degree, n (%)		10 (10)	9 (11)	6 (9)	0.932
	PhD	5 (5)	5 (6)	4 (6)	0.941
	DDM/DDS/DMD	4 (4)	4 (5)	0 (0)	0.854
	Other	0 (0)	0 (0)	0 (0)	---
Number of advanced degrees, median (range)		0 (0-2)	0 (0-1)	0 (0-2)	0.997
Residency program attended Doximity reputation rank, median (IQR)		7 (4-16)*	16 (6-33)	28 (9-53)	<0.001
Residency program attended Doximity research rank, median (IQR)		8 (4-15)*	22 (8-41)	27 (12-55)	<0.001
International residency attended, n (%)		6 (6)	13 (16)*	4 (6)	0.039
US residency attended, n (%)					0.307
	Integrated	12 (12)	11 (14)	8 (12)	
	Independent	81 (84)	59 (75)	59 (84)	
	N/A	4 (4)	9 (11)	3 (4)	
Fellowships, n (%)		70 (72)	57 (74)	46 (66)	0.508
	Microsurgery	17 (18)	10 (13)	8 (12)	
	Hand	18 (19)	27 (34)	11 (16)	
	Craniofacial	28 (29)	14 (18)	17 (24)	
	Aesthetic	0 (0)	2 (3)	3 (4)	
	Burn	2 (2)	3 (4)	3 (4)	
	Peripheral nerve	1 (1)	3 (4)	1 (1)	
	Other	4 (4)	5 (6)	3 (4)	
	None	34 (35)	25 (32)	28 (40)	
International fellowship, n (%)		11 (11)	7 (9)	6 (9)	0.859
Research fellowship, n (%)		15 (15)	21 (27)	12 (17)	0.210
Number of fellowships, median (range)		1 (0-3)	1 (0-3)	1 (0-3)	0.204
Number of years in practice, median (range)		22 (5-46)	26 (8-51)	27 (7-50)	0.102
Department faculty, n (%)		26 (27)	25 (32)	10 (14)*	0.036
Endowed status, n (%)		28 (29)	18 (23)	9 (13)*	0.041
Residency director, n (%)		7 (7)	9 (12)	13 (19)	0.089
Fellowship director, n (%)		15 (15)	10 (13)	5 (7)	0.333
	Aesthetic	3 (3)	1 (1)	1 (1)	
	Craniofacial	5 (5)	4 (5)	2 (3)	
	Microsurgery	4 (4)	1 (1)	2 (3)	
	Hand	2 (2)	4 (5)	0 (0)	
	None	82 (85)	67 (87)	65 (93)	
Chief/chair, n (%)		20 (21)	22 (28)	30 (43)*	<0.001
Former chief/chair, n (%)		5 (5)	8 (10)	2 (3)	0.138
Journal editorial board, n (%)		35 (36)	30 (38)	18 (26)	0.274
ACGME board member, n (%)		5 (5)	3 (4)	1 (1)	0.482
Officer/director of ABPS, n (%)		22 (23)	19 (25)	11 (16)	0.385
President of national society/association, n (%)		27 (28)	22 (29)	9 (13)	0.053
Number of presidencies of national society/association, median (range)		0 (0-6)*	0 (0-5)	0 (0-4)	0.042
President of regional society/association, n (%)		11 (11)	9 (12)	7 (10)	0.947
Number of publications, median (range)		114 (1-920)*	64 (2-625)*	36 (0-185)*	<0.001
Number of first-author publications, median (range)		19 (0-159)	14 (0-115)	8 (0-41)*	<0.001
Number of last-author publications, median (range)		48 (0-542)*	23 (1-188)*	12 (0-124)*	<0.001
Number of first and last-author publications, median (range)		65 (0-701)*	36 (2-303)*	23 (0-136)*	<0.001
H-Index, median (range)		26 (1-104)*	20 (0-81)*	15 (0-43)*	<0.001
Number of citations, median (range)		2,611 (1-42,005)*	1,487 (0-24,114)*	884 (0-5,447)*	<0.001
NIH funded, n (%)		33 (34)*	12 (15)*	4 (6)*	<0.001
Number of NIH grants, median (range)		0 (0-89)	0 (0-33)	0 (0-10)*	<0.001
Total NIH funding, median (range)		0 (0-42,232,565)	0 (0-13,597,384)	0 (0-2,971,116)*	<0.001
AAPS award, n (%)		27 (28)*	9 (11)*	5 (7)*	0.001
Number of distinct AAPS awards, median (range)		0 (0-4)	0 (0-4)	0 (0-3)*	0.001

Following multivariate analysis, plastic surgeons were more likely to move laterally from a professor at a T3 program to a T2 program if they were a chief/chair (OR: 3.05, 95% CI: 1.56, 5.97; AUC: 0.42; p = 0.001). Plastic surgeons were more likely to move laterally from a professor at a T3 program to a T2 program and T1 program with a greater number of last-author publications (OR: 1.02, 95% CI: 1.01, 1.03; AUC: 0.78; p < 0.001), if they received any AAPS award (OR: 6.46, 95% CI: 1.09, 38.46; AUC: 0.79; p = 0.039), and a greater number of AAPS awards (OR: 4.62, 95% CI: 1.41, 15.23; AUC: 0.79; p = 0.012).

Promotion Ladder of Academic Plastic Surgery

**Figure 1:**
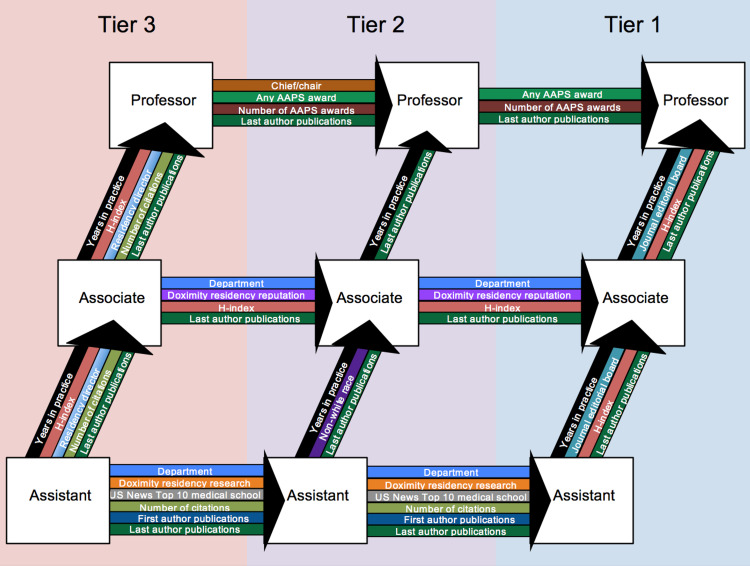
Promotion Ladder of Academic Plastic Surgery. AAPS: American Association of Plastic Surgeons

Six multivariate regression models were combined to synthesize the *Promotion Ladder of Academic Plastic Surgery* from three Doximity program tiers (Figure 1).

Independent predictors of promotion from assistant to associate to professor within a program tier correspond to bottom-to-top advancement up the promotion ladder. Independent predictors of promotion from T3 to T2 to T1 programs correspond to left-to-right advancement across the promotion ladder.

## Discussion

We evaluated objective metrics between academic plastic surgeons from three Doximity program tiers to determine predictors and guide future and/or current surgeons seeking promotion from assistant professors to associate professors to full professors. Furthermore, we evaluated academic plastic surgeons at the same professorship level from three different tiered programs to guide those seeking lateral movement between programs. Independent predictors following comparisons were used to synthesize the Doximity-tiered *Promotion Ladder of Academic Plastic Surgery*.

Increasing the number of last-author publications was the most impactful method that predicted promotion at every intersection of the promotion ladder. By increasing the number of last-author publications, a plastic surgeon can advance up and across the promotion ladder. Traditionally, first authorship designates a junior team member who collected data and synthesized the manuscript, while last authorship designates a senior faculty member who contributed intellectual property through experience [6]. In addition, the last author often carries the prestige and responsibility of the corresponding author. Corresponding authors are responsible for accurate manuscript content, criticisms, and addressing any comments [6]. In contrast, first-author publications only predicted promotion for assistant professors advancing across the ladder from T3 to T1 programs. The prestige and responsibility associated with last authorship may indicate a higher level of academic maturity than first authorship with subsequent advancement up and across the promotion ladder.

H-indexes are bibliometric measurements used to assess the quality of publications [1,2,7-10]. Higher h-indexes have previously had the greatest associations with tenure promotion following bibliometric comparisons among the h-index, g-index, hc-index, and number of peer-reviewed publications [2]. Others found the h-index, I-10 index, total number of publications, and total number of citations all had similar correlations with academic rank [1]. Our observations indicate h-indexes were predictors of promotion at T1 programs, T3 programs, and for associate professors moving laterally from T3 to T1 programs, while the number of citations was a predictor of promotion at T3 programs and for assistant professors moving laterally from T3 to T1 programs.

Dedicating more years in practice predicted promotion up the ladder within all program tiers. Years in practice were measured from the time of first becoming an attending physician to the 2020-2021 academic calendar year. More years in practice increase the opportunities of achieving excellence in all five classic principles that define an academic surgical career [11,12]. We were not able to assess the impact of duration at a single institution. Some academic surgeons remain at an institution for their entire career, while others seek promotion by leaving an institution. Only static variables in 2020 were assessable, but not specific reasons for promotion.

Plastic surgery programs designated as departments have the autonomy to eliminate bureaucracy and layers of administration, directly engage in educational decisions with the dean of the medical school, implement educational plans, and use surplus revenues to recruit and maintain faculty [13]. Departments predicted lateral movement for assistant and associate professors across the ladder from T3 to T1 programs. At the status level of full professorship, receiving any AAPS award and a greater number of AAPS awards predicted lateral movement for full professors across the ladder from T3 to T1 programs. Depending on a surgeon’s current level of professorship, seeking programs designated as departments or applying for AAPS awards may provide opportunities for lateral movement across the promotion ladder.

Institutional programs were stratified into three tiers by Doximity research rankings. Doximity determines rankings by current resident and recent alumni satisfaction data, reputation data, and objective data [4]. Satisfaction data were determined by survey results from graduates within the past 15 years or current residents. Reputation data were determined by peer nominations from board-certified plastic surgeons. Objective data were determined by the currently available program, resident, and board certification metrics [4]. Although Doximity rankings may be subjected to criticism, these were the most universally accepted publicly available data. A three-tiered Doximity approach was selected to maintain study power for statistical comparisons while differentiating more stringent requirements between different plastic surgery programs. Data were evaluated from the 2020-2021 academic calendar year. A single-year assessment limited comparisons over years to determine yearly productivity for each academic surgeon or time intervals to promotion over a career. Only 851 plastic surgeons had publicly available professorship data and were eligible for inclusion from the sample of 951 plastic surgeons. Four programs were not Doximity ranked (University of Alabama; Mayo Clinic, Florida; Medical College of Georgia; University of Minnesota). These programs were added as T3 programs. Pediatric fellowships were not considered their own fellowship. They were combined with either craniofacial or hand depending on the primary focus of the fellowship. Publicly available leadership in regional and national societies/associations was limited to past presidents. Assessing other board leadership positions and overall member statuses would have provided greater insight into the impact of regional and national society/association affiliations. We were not able to assess the classic principle of clinical productivity. RVUs and incomes were not publicly available data, limiting assessments of the importance of case volume on an academic surgical career. In addition, we were not able to assess the impact of mentorship on promotion. Mentors impact career choices of students, residents, fellows, and junior faculty [14]. Due to individual variability of faculty and different career goals, the discrimination measured by AUCs ranged from 0.23 to 0.86. Not all academic plastic surgeons are seeking promotion. Full-time plastic surgery faculty affiliated with United States training programs were evaluated, limiting the generalizability of promotion predictors to the United States.

Academic plastic surgeons should follow institution-specific promotion criteria. While we realize our model may not fit all institutions, our comprehensive data collection and rigorous methodology provided a generalized framework to assess independent predictors associated with promotion and lateral movement in academic plastic surgery from the 2020-2021 academic calendar year. Independent promotion predictors provided evidence to synthesize the Doximity-tiered *Promotion Ladder of Academic Plastic Surgery*.

## Conclusions

Academic plastic surgeons were more likely to be promoted from assistant to associate and professor at T1 programs with more years in practice, being on a journal editorial board, a greater number of last-author publications, and higher h-indexes. Promotion from assistant to associate was more likely at T2 programs if surgeons were non-white. Surgeons were more likely to be promoted to associate and professor at T2 programs with more years in practice and a greater number of last-author publications. Surgeons were more likely to be promoted to associate and professor at T3 programs with more years in practice, as a residency director, a greater number of last-author publications, higher h-indexes, and a greater number of citations. Assistant professors at T3 programs were more likely to move laterally between T2 and T1 programs if they attended a Top 10 US News medical school, attended a more favorable Doximity-ranked research program, with a greater number of first-author publications, a greater number of last-author publications, a greater number of citations, and if plastic surgery was a department at T2 and T1 programs. Associate professors at T3 programs were more likely to move laterally between T2 and T1 programs if they attended a more favorable Doximity-ranked reputation program, with a greater number of last-author publications, higher h-indexes, and if plastic surgery was a department at T2 and T1 programs. Professors at T3 programs were more likely to move laterally to T2 programs if they were a chief/chair. Professors at T3 programs were more likely to move laterally between T2 and T1 programs with a greater number of last-author publications, if they received any AAPS award, and a greater number of AAPS awards. These Independent predictors were used to provide evidence and synthesize the Doximity-tiered *Promotion Ladder of Academic Plastic Surgery*.

## Appendices

Appendix 1: Doximity research-ranked plastic surgery program tiers

Supplemental Digital Content 1. Plastic Surgery Program Tiers. Integrated/independent plastic surgery programs categorized into three tiers by Doximity research rankings

Tier 1 (1-20): Baylor College of Medicine; University of Michigan; University of Pittsburgh; Johns Hopkins University; University of California, Los Angeles (UCLA); Northwestern University; Duke University; New York University (NYU); University of Pennsylvania; Georgetown; University of Washington (UW); Stanford; New York Presbyterian Hospital/Weill Cornell; University of Chicago; Harvard-Brigham and Women’s Hospital/Massachusetts General Hospital; University of Texas Southwestern (UTSW); University of California, San Francisco (UCSF); University of California, Irvine; Case Western Reserve University; University of Rochester.

Tier 2 (21-50): University of California, Davis; University of Virginia; Cleveland Clinic Foundation, Ohio; University of Missouri; University of Kentucky; University of North Carolina (UNC); Brown University; Loma Linda University; Emory University; Nassau University Medical Center/Long Island Plastic Surgical Group/Stony Brook Medicine; Southern Illinois University; Saint Louis University (SLU); University of Wisconsin; Rutgers University; Washington University in St. Louis (WashU); Medical College of Wisconsin; Mayo Clinic, Rochester; Mayo Clinic, Phoenix/Scottsdale; University of Southern California (USC); Ohio State University; University of Texas Medical Branch, Galveston (UTMB); University of Miami; Icahn School of Medicine at Mount Sinai; Yale; Wake Forest; Spectrum Health/Michigan State University; Indiana University (IU); University of Florida (UF); Penn State Health Milton S. Hershey Medical Center; University of Kansas.

Tier 3 (>50/not ranked): University of South Florida (USF); University of California, San Diego (UCSD); Lehigh Valley Health Network (LVHN); Oregon Health and Science University (OHSU); University of Cincinnati (UC); Texas A&M/Baylor Scott & White Medical Center-Temple; Albany Medical Center; University of Nevada Las Vegas (UNLV); Virginia Commonwealth University (VCU); University of Massachusetts; Medical University of South Carolina (MUSC); Louisiana State University (LSU); University of Mississippi; Montefiore Medical Center/Albert Einstein; University of Utah; Hofstra/Northwell Health; Wright State University; Loyola University of Chicago; Lahey Clinic; University of Colorado; Farmington Hills and Royal Oak; Beth Israel Deaconess Medical Center/Harvard (BIDMC); Carilion Clinic/Virginia Tech; Cleveland Clinic Foundation, Florida; Cooper University Hospital; Dartmouth-Hitchcock; Detroit Medical Center/Wayne State; Henry Ford Hospital/Wayne State; Geisinger Health System; Houston Methodist Hospital; Prisma Health-Midlands/USC; Rush University Medical Center; Summa Health System/NEOMED; Temple University Hospital; Tulane University/Ochsner Clinic; University of Illinois Chicago (UIC); University of Louisville; University of Nebraska; University of New Mexico (UNM); University of Tennessee Health Science Center (UTHSC); University of Tennessee Health Science Center, Chattanooga; University of Texas, Houston; University of Texas, San Antonio; Vanderbilt; West Virginia University; University of Alabama (UAB); Mayo Clinic, Florida; Medical College of Georgia; University of Minnesota.

Appendix 2: Variables analyzed

Variables included physician demographics (race, sex), current faculty academic institutions, medical degree backgrounds [Doctor of Medicine (MD), International Medical Graduate (IMG), Doctor of Osteopathic Medicine (DO)], advanced degrees [Master of Arts (MA), Master of Science (MS), Master of Business Administration (MBA), Master of Health Science (MHS), Master of Public Health (MPH), Doctor of Philosophy (PhD), Doctor of Dental Medicine/Doctor of Dental Surgery/Doctor of Medicine in Dentistry (DDM/DDS/DMD), other], residency training (integrated or independent), fellowship training (microsurgery, hand, craniofacial, aesthetic, burn, peripheral nerve, other), number of years in practice, program division or department status, residency and fellowship directorship (aesthetic, craniofacial, microsurgery, hand), editorial board status (Plastic and Reconstructive Surgery, Plastic and Reconstructive Surgery-Global Open, Annals of Plastic Surgery, Journal of Craniofacial Surgery, Cleft Palate-Craniofacial Journal, Journal Plastic, Reconstructive, & Aesthetic Surgery, Microsurgery, Journal of Reconstructive Microsurgery, Aesthetic Surgery Journal, Journal of Hand Surgery, and Hand), ACGME board member, officer/director of the ABPS, regional society/association presidencies [California Society of Plastic Surgeons (CSPS), Southeastern Society of Plastic and Reconstructive Surgeons (SESPRS), Mountain West Society of Plastic Surgery (MWSPS), Ohio Valley Society of Plastic Surgeons (OVSPS), Midwestern Society of Plastic Surgeons (MAPS), Northwest Society of Plastic Surgeons (NWSPS), Northeastern Society of Plastic Surgeons (NESPS)], national society/association presidencies [American Burn Association (ABA), American Council of Academic Plastic Surgeons (ACAPS), American College of Surgeons (ACS), American Society for Reconstructive Microsurgery (ASRM), American Association for Hand Surgery (AAHS), American Society for Surgery of the Hand (ASSH), American Society for Peripheral Nerve (ASPN), Plastic Surgery Research Council (PSRC), American Society of Maxillofacial Surgeons (ASMS), American Society of Craniofacial Surgeons (ASCFS), American Society for Aesthetic Plastic Surgery (ASAPS), Plastic Surgery Foundation (PSF), American Society of Plastic Surgeons (ASPS), American Association of Plastic Surgeons (AAPS), American Society of Transplant Surgeons (ASTS)], research metrics [total number of publications, number of first and last-author publications, Hirsch or h-indexes, number of citations (Scopus; Reed Elsevier, London, United Kingdom)], National Institute of Health (NIH) grant funding (number of NIH grants, total NIH funding), AAPS awards (Clinician of the Year, The Honorary Award, Academic Scholarship, Distinguished Fellows, Research Achievement Award, Leonard R. Rubin Award, James Barrett Brown Award), and number of AAPS awards.

## References

[1] Susarla SM, Lopez J, Swanson EW (2015). Are quantitative measures of academic productivity correlated with academic rank in plastic surgery? A National Study. Plast Reconstr Surg.

[2] Gast KM, Kuzon WM Jr, Waljee JF (2014). Bibliometric indices and academic promotion within plastic surgery. Plast Reconstr Surg.

[3] von Elm E, Altman DG, Egger M, Pocock SJ, Gøtzsche PC, Vandenbroucke JP (2014). The Strengthening the Reporting of Observational Studies in Epidemiology (STROBE) Statement: guidelines for reporting observational studies. Int J Surg.

[4] (2020). Doximity: Plastic Surgery (Integrated) Residency Programs. https://residency.doximity.com/specialties/56-plastic-surgery-integrated.

[5] Beasley MT, Schumacker R (1995). Multiple regression approach to analyzing contingency tables: post hoc and planned comparison procedures. J Exp Educ.

[6] Grinsell D, Jovic TH, Saravolac V, Whitaker IS (2020). Authorship in surgical articles. J Plast Reconstr Aesthet Surg.

[7] Hu J, Gholami A, Stone N, Bartoszko J, Thoma A (2018). An evaluation of h-index as a measure of research productivity among Canadian academic plastic surgeons. Plast Surg (Oakv).

[8] Hirsch JE (2005). An index to quantify an individual's scientific research output. Proc Natl Acad Sci U S A.

[9] Svider PF, Pashkova AA, Choudhry Z (2013). Comparison of scholarly impact among surgical specialties: an examination of 2429 academic surgeons. Laryngoscope.

[10] Therattil PJ, Hoppe IC, Granick MS, Lee ES (2016). Application of the h-Index in academic plastic surgery. Ann Plast Surg.

[11] Chen JT, Girotto JA, Kitzmiller WJ (2014). Academic plastic surgery: faculty recruitment and retention. Plast Reconstr Surg.

[12] Waljee JF, Chung KC (2014). Discussion: academic plastic surgery: faculty recruitment and retention. Plast Reconstr Surg.

[13] Guyuron B (2008). Academic plastic surgery: division or department?. Aesthet Surg J.

[14] Wagner IJ, Hultman CS (2013). Elevation: developing a mentorship model to raise the next generation of plastic surgery professionals. Ann Plast Surg.

